# Evidence for Phenotypic Plasticity in Aggressive Triple-Negative Breast Cancer: Human Biology Is Recapitulated by a Novel Model System

**DOI:** 10.1371/journal.pone.0045684

**Published:** 2012-09-25

**Authors:** Nicholas C. D’Amato, Julie H. Ostrander, Michelle L. Bowie, Christopher Sistrunk, Alexander Borowsky, Robert D. Cardiff, Katie Bell, Lawrence J. T. Young, Karl Simin, Robin E. Bachelder, Jeff Delrow, Alyssa Dawson, Lisa D. Yee, Krzysztof Mrózek, Timothy M. Clay, Takuya Osada, Victoria L. Seewaldt

**Affiliations:** 1 Department of Pharmacology and Cancer Biology, Duke University, Durham, North Carolina, United States of America; 2 Department of Medicine, University of Minnesota Masonic Cancer Center, Minneapolis, MInnesota, United States of America; 3 Department of Medicine, Duke University Medical Center, Durham, North Carolina, United States of America; 4 Center for Comparative Medicine, University of California Davis, Davis, California, United States of America; 5 Department of Cancer Biology, University of Massachusetts Medical School, Worcester, Massachusetts, United States of America; 6 Department of Pathology, Duke University, Durham, North Carolina, United States of America; 7 Genomics Resource, Fred Hutchinson Cancer Research Center, Seattle, Washington, United States of America; 8 Comprehensive Cancer Center, The Ohio State University, Columbus, Ohio, United States of America; 9 Department of Surgery, Duke University Medical Center, Durham, North Carolina, United States of America; Massachusetts General Hospital, United States of America

## Abstract

Breast cancers with a basal-like gene signature are primarily triple-negative, frequently metastatic, and carry a poor prognosis. Basal-like breast cancers are enriched for markers of breast cancer stem cells as well as markers of epithelial-mesenchymal transition (EMT). While EMT is generally thought to be important in the process of metastasis, *in vivo* evidence of EMT in human disease remains rare. Here we report a novel model of human triple-negative breast cancer, the DKAT cell line, which was isolated from an aggressive, treatment-resistant triple-negative breast cancer that demonstrated morphological and biochemical evidence suggestive of phenotypic plasticity in the patient. The DKAT cell line displays a basal-like phenotype *in vitro* when cultured in serum-free media, and undergoes phenotypic changes consistent with EMT/MET in response to serum-containing media, a unique property among the breast cancer cell lines we tested. This EMT is marked by increased expression of the transcription factor Zeb1, and Zeb1 is required for the enhanced migratory ability of DKAT cells in the mesenchymal state. DKAT cells also express progenitor-cell markers, and single DKAT cells are able to generate tumorspheres containing both epithelial and mesenchymal cell types. *In vivo*, as few as ten DKAT cells are capable of forming xenograft tumors which display a range of epithelial and mesenchymal phenotypes. The DKAT model provides a novel model to study the molecular mechanisms regulating phenotypic plasticity and the aggressive biology of triple-negative breast cancers.

## Introduction

Breast cancer is a heterogeneous disease that exhibits a wide range of clinical behaviors, prognoses, and histologies. Gene expression profiling of patient samples initially identified five breast cancer subgroups (luminal A and B, Her2 amplified, normal-like, and basal-like subtypes), each with a distinct molecular signature [1]–[3]. Tumors with a basal-like gene signature are primarily triple-negative (ER−/PR−/Her2wt), frequently occur in young African American women and *BRCA1* mutation carriers, and carry the worst prognosis [2]–[5]. While some triple-negative breast cancers respond to treatment, a subset are highly invasive and metastatic and do not respond to chemotherapy or radiation [6]. Recent studies have shown that triple-negative breast cancers are enriched for markers of epithelial-mesenchymal transition (EMT), a process generally thought to be important in the metastatic cascade [7], [8]. Other recent work has demonstrated a link between EMT and stem cell-like characteristics in mammary epithelial cells, and that an EMT and stem cell-like gene expression signature is found in residual breast cancer cells following chemotherapy [9], [10]. Collectively, these studies suggest that epithelial-mesenchymal plasticity may be important for the highly aggressive subset of triple-negative breast cancers.

Epithelial-mesenchymal transition is a part of normal physiological processes, including embryogenesis and wound healing, in which cells of epithelial origin lose epithelial characteristics and polarity and acquire a mesenchymal phenotype associated with increased migratory behavior [11]–[14]. Activation of an EMT-like program in cancer cells *in vitro* similarly results in increased cell migration and invasion as well as increased resistance to apoptosis [7], [11]. At the molecular level, EMT is characterized by 1) loss of expression of membranous E-cadherin, claudins, and occludins, 2) increased expression of mesenchymal markers including vimentin and smooth muscle actin, 3) acquisition of a spindle-like morphology, and 4) cytoskeleton reorganization [12], [14]. The reverse process, mesenchymal-epithelial transition (MET), is characterized by a loss of expression of mesenchymal markers and restoration of epithelial markers and morphology [13], [15].

The similarities between the developmental EMT events and the process of tumor cell dissemination, in which cells lose contact with the primary tumor and invade into the normal host tissue and blood vessels, has lead to the hypothesis that EMT is an important part of the metastatic cascade (10–13). However, there is difficulty in identifying EMT in human breast cancer because the full sequence of events that have come to define EMT *in vitro* are not commonly observed *in vivo* (29), and metastases commonly have an epithelial phenotype similar to the primary tumor. In order to reconcile the observations of breast cancer pathologists with *in vitro* studies of breast cancer cell lines, it has been proposed that EMT in the human tumor setting may be transient and reversible, and that this phenotypic plasticity may be a key determinant of metastatic potential [7], [15], [16].

The study of plasticity in human breast cancer is currently limited by a lack of appropriate models that can reversibly transition from the epithelial to mesenchymal state. Here we report the isolation and characterization of the DKAT cell line, a novel model of triple-negative breast cancer that was isolated from a rapidly progressing, treatment-resistant, metastatic human breast cancer. *In vitro*, DKAT cells are able to undergo phenotypic changes consistent with EMT/MET in response to altered culture conditions and express markers of breast cancer stem cells, suggesting inherent phenotypic plasticity. Similar to the human tumor from which the cell line was isolated, in xenograft experiments DKAT cells form heterogeneous tumors with regions of both epithelial and mesenchymal characteristics. The DKAT cell line provides a new model to investigate the molecular mechanisms of phenotypic plasticity in aggressive triple-negative breast cancers.

## Results

### Evidence Suggestive of Epithelial-mesenchymal Plasticity in a Human Triple-negative Breast Cancer

The DKAT cell line was derived from the malignant pleural effusion of a 35-year-old Caucasian woman with no family history of breast or ovarian cancer. The patient initially presented with a 4 cm ER/PR(−/−), HER2/Neu-wt, CK5(+), EGFR(+), lymph node-negative breast cancer (T2N0M0). At the sixth cycle of cyclophosphamide, methotrexate, and 5-fluorouracil, seven months from the initial diagnosis, recurrent cancer was detected within the chest wall radiation field. At ten months from initial diagnosis, metastases to the lung, pleura, liver, and bone were observed. At eleven months, the patient developed pancytopenia; a bone marrow biopsy revealed multiple foci of tumor cells within the bone marrow. At this time the tumor was not responsive to taxotere and navelbine. At twelve months from initial diagnosis the patient died from pancytopenia and rapid disease progression.

**Figure 1:**
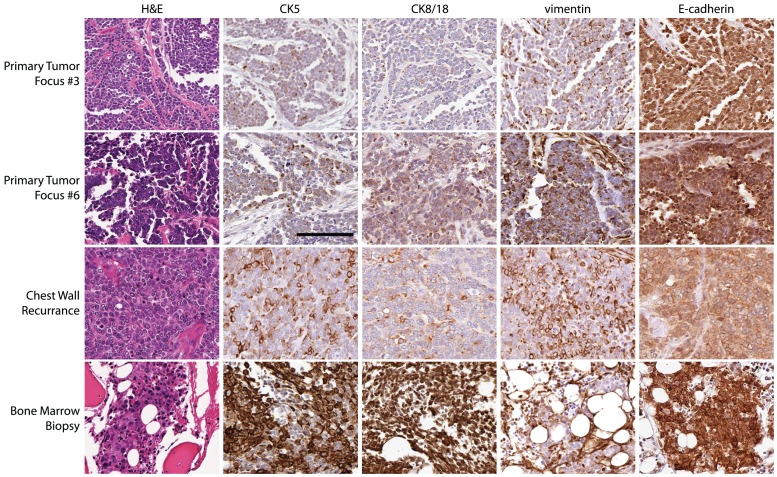
Human breast cancer specimen displays morphological and biochemical evidence suggestive of EMT and MET. Images of the patient’s primary tumor focus #3 (top row), focus #6 (second row), chest wall recurrence (third row), and bone marrow biopsy (bottom row) showing H&E staining and immunohistochemistry for CK5, CK8/18, vimentin, and E-cadherin.

The primary breast cancer surgical specimen demonstrated a range of phenotypes. Six widely separated microfocal neoplastic lesions were identified that exhibited varying morphological and immunohistochemical features (summarized in Table 1). Primary Tumor Foci #1-5 stained strongly for the epithelial marker E-cadherin and displayed only scattered weak staining for CK8/18, CK5, and the mesenchymal marker vimentin (Figure 1, top row). In contrast, Primary Tumor Focus #6 was strongly positive for vimentin and CK5 as well as CK8/18 and E-cadherin (Figure 1, second row). Similar to Primary Tumor Focus #6, the chest wall recurrence contained solid sheets of malignant cells that were positive for vimentin, CK5, and CK8/18, but E-cadherin staining was notably less intense (Figure 1, third row). The staining pattern of the malignant cells found in the bone marrow, however, was similar to the epithelial staining pattern observed in Primary Tumor Foci #1-5 with cells staining strongly for CK5, CK8/18 and membranous E-cadherin, but not vimentin (Figure 1, bottom row). These observations show that the original primary tumor contained both epithelial and mesenchymal regions, the chest wall recurrence displayed increased expression of mesenchymal markers but still retained epithelial markers, suggesting an intermediate phenotypic state, while the metastatic lesions in the bone strongly expressed epithelial markers. A complete list of markers expressed in the primary tumor, chest wall recurrence, and bone marrow metastasis is provided in Table 1. While these observations could be the result of clonal heterogeneity within the tumor, another possible explanation is they represent areas of progression through a transient EMT/MET. Therefore, we tested the cell line derived from this patient for phenotypic plasticity in culture.

**Table 1:** Characterization of human primary tumor, chest wall recurrence and bone marrow metastasis.

Marker	Primary Foci #1-5	Primary Foci #6	Chest Wall Recurrence	Bone Marrow Metastasis
ER	(−)	(−)	nt	nt
PR	(−)	(−)	nt	nt
HER2/neu	Not amplified	Not amplified	nt	nt
CK5	(+)	(++)	(++)	(+++)
CK14	(+)	(+)	(+)	(+)
CK8/18	(+)	(++)	(+)	(+++)
E-cadherin				
membrane	(++)	(+)	(+)	(+++)
cytoplasmic	(++)	(++)	(+)	(++)
Vimentin	(+)	(+++)	(+++)	(+)

Qualitative evaluation based on *in situ* hybridization for Her2, and based on immunohistochemistry for all other markers.

### *In vitro* Characteristics of DKAT Cell Line Reflect the Primary Tumor

**Figure 2:**
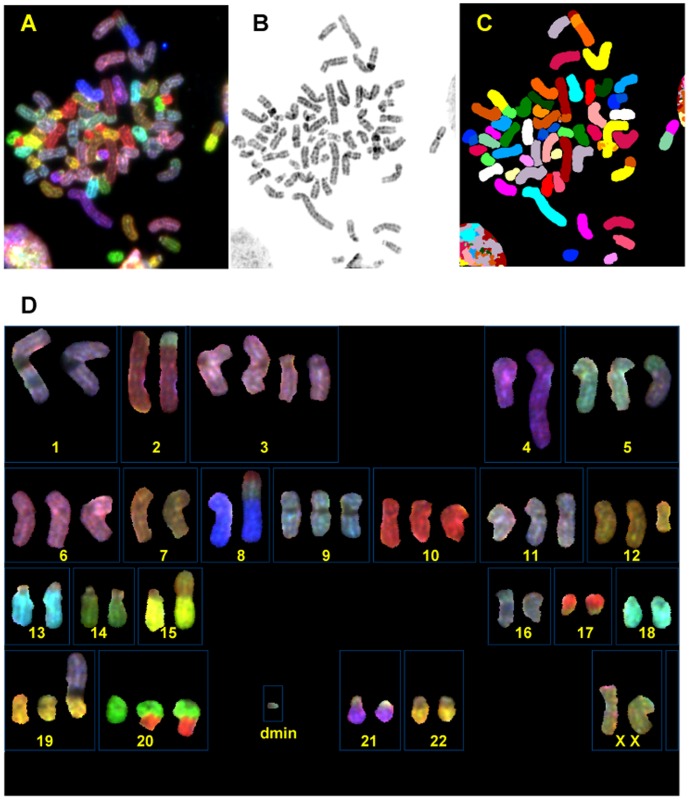
DKAT cell line karyotype. A representative mitotic DKAT cell from sideline 1 is shown. **A.** Metaphase cell with chromosomes shown in SKY display colors. **B.** Inverted and contrast-enhanced DAPI image of the same metaphase cell. **C.** The same metaphase cell with chromosomes shown in spectra-based classification colors. **D.** Spectral karyotype of the same metaphase cell shown in SKY display colors.

Cells isolated from the patient’s malignant pleural effusion rapidly adapted to tissue culture conditions. Forty-eight hours after isolation (passage 1) DKAT cells began proliferating in culture with a doubling time of approximately 24 hours (data not shown). The DKAT cell line has been maintained continuously in culture for >70 passages. Twenty-five metaphase DKAT cells (passage 3) were subjected to spectral karyotyping (SKY) analysis, and an additional 9 cells were G-banded and karyotyped (Figure 2). The modal chromosome number was 56. In addition to 29 cells with the hyperdiploid chromosome number, 3 cells had hypopentaploid chromosome number (104–109 chromosomes) and 2 cells had either 117 or 127 chromosomes. Among the predominant hyperdiploid cells, two clones, a stemline and a sideline 1, were identified. The only difference between the stemline and sideline 1 is the presence of one double minute (dmin) in a sideline. Because of its small size, the origin of this double minute could not be identified with confidence. Sideline 2 is near-tetraploid and represents a doubling of the stemline, with some random chromosome losses and a few non-clonal aberrations in the cells, and sideline 3 is a composite karyotype of 2 cells with the highest chromosome numbers.

Aggressive triple-negative breast cancers frequently contain mutations in the *TP53* and *PIK3CA* genes [2], [17], [18]. Sequencing of exons 5–9 of the *TP53* gene in DKAT cells identified a single point mutation in exon 8 at codon 273 (CGT > CAT). This is a missense mutation previously described in the MDA-MB-468 breast cancer cell line, and reported to be deleterious to p53 function [19], [20]. In breast tumors, *PIK3CA* mutations have been found to frequently occur in exons 9 and 20 [21]. Sequencing of *PIK3CA* exons 9 and 20 from DKAT genomic DNA revealed no mutations within the coding region of these two exons.

**Figure 3:**
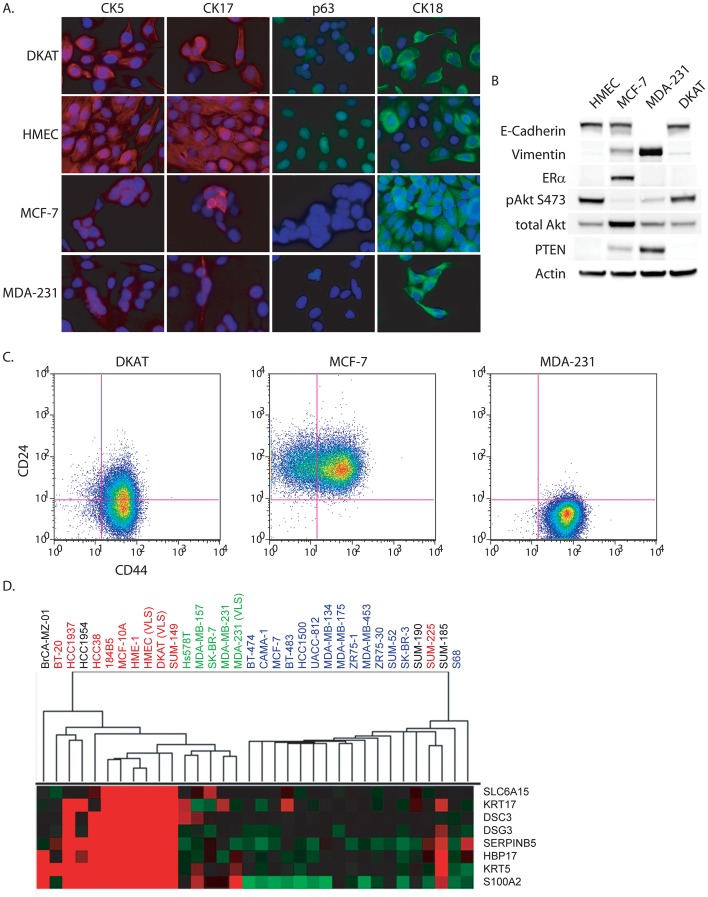
DKAT cells express markers of basal epithelial cells. A. Immunofluorescence for CK5, CK17, p63 and CK18 in DKAT, immortalized HMEC, MCF-7, and MDA-MB-231 cells. **B**. Western blotting of total cell lysates from HMEC, MCF-7, MDA-MB-231, and DKAT cells grown in MEGM. **C.** FACS analysis of DKAT-MEGM, MCF-7 and MDA-MB-231 cells stained for CD44 and CD24. **D.** Results from DKAT, MDA-MB-231, and HMEC Affymetrix HG-U133 2+ arrays were analyzed by unsupervised hierarchical clustering with a published data set [28]. Cell lines are labeled by breast cancer subtype; luminal-like (blue); basal-like (red); mesenchymal-like (green); unknown subtype (black). Displayed is an expanded view of selected gene cluster containing basal-specific gene expression. The full gene cluster is provided as Figure S1.

A variety of techniques were used to test DKAT cells for baseline expression of proteins associated with triple-negative breast cancers and an aggressive phenotype including markers of EMT, progenitor-like cell markers, and markers of PI3K/AKT pathway activation. A summary of baseline DKAT expression data is provided in Table 2. Immunohistochemical analysis showed that DKAT cells did not stain for ER/PR but stained positively for EGFR, and fluorescence *in situ* hybridization demonstrated diploid copy number of *HER2*, consistent with the triple-negative primary human tumor (data not shown). Immunofluorescence and western blotting showed that DKAT cells express other markers consistent with basal mammary epithelial cells, including CK5 and CK17, p63, and E-cadherin, which was found localized to the membrane and throughout the cytoplasm (Figure 3A). DKAT cells also express the luminal epithelial cell marker CK18, although all other cell lines tested also expressed CK18 including the mesenchymal MDA-MB-231 line [22], [23]. Western blot analyses demonstrated that DKAT cells express a high level of Akt phosphorylated at Serine 473, a common feature of triple-negative breast cancers (Figure 3B) [24]. While we did not observe mutations in PIK3CA exons 9 or 20, Akt phosporylation at Ser 473 may be explained by the relatively low levels of PTEN in the DKAT cell line (Figure 3B). FACS analysis of the cell surface markers CD44 and CD24 revealed that the DKAT cell line has a high percentage of cells of the CD44^+^/CD24^−/low^ phenotype reported to be enriched for tumorigenic and stem-like cell activity [25], [26] and more recently associated with cells that have undergone EMT [10], [27] (Figure 3C). Taken together, these observations demonstrate that DKAT cells exhibit baseline expression of basal and luminal epithelial markers, phosphorylation of AKT-Serine 473, and express progenitor-like cell markers.

**Table 2:** Summary of DKAT marker expression (MEGM/SCGM).

	DKAT line	
Marker	MEGM media	SCGM media
ER	(−)	(−)
PR	(−)	(−)
HER2	Not amplified	Not amplified
EGFR	(+)	(+)
CK5	(++)	(−)
CK14	(−)	(−)
CK17	(++)	(+/−)
CK8	(++)	(+/−)
CK18	(++)	(+/−)
E-cadherin		
membrane	(+)	(−)
cytoplasmic	(+)	(+)
vimentin	(+/−)	(+++)
p63	(+)	(−)
snail-1	(++)	(++)
snail-2/slug	(+)	(+)
claudin-1	(+)	(−)
claudin-4	(+)	(−)
occludin	(+)	(−)
Akt	(+)	(+)
pAkt-Ser473	(+++)	(++)
PTEN	(−)	(+)
p53	mutant	mutant
PI3K	wt	wt

Qualitative evaluation based on immunofluorescence or western blot of DKAT cells grown in MEGM or following 14 days in SCGM media.

In order to assess the relationship of DKAT cells to published breast cancer subtypes, we performed unsupervised hierarchical clustering of DKAT transcript expression. Comparing our experimental data with a previously published analysis of breast cancer cell lines [28], we found that the DKAT gene expression profile clustered with a number of basal-type cell lines (Figure 3D, Figure S1). DKAT cells cluster most closely with SUM-149 cells, but also with 184B5, MCF-10A, and HMEC cells. Parallel analysis of control MDA-MB-231 and HMEC transcripts showed a high correlation to previously published microarray data (r = 0.70 and r = 0.84, respectively), validating the effectiveness of combining these two data sets.

### Evidence for Reversible EMT/MET *in vitro*

DKAT cells were derived from a breast cancer that exhibited a diverse range of phenotypes, but it is unclear whether this phenotypic diversity was the result of 1) outgrowth or evolution of specialized subpopulations of cells within the primary tumor or 2) phenotypic plasticity inherent within the tumor cells. We tested DKAT cells for evidence of phenotypic plasticity by altering culture conditions and testing for *in vitro* EMT/MET.

**Figure 4:**
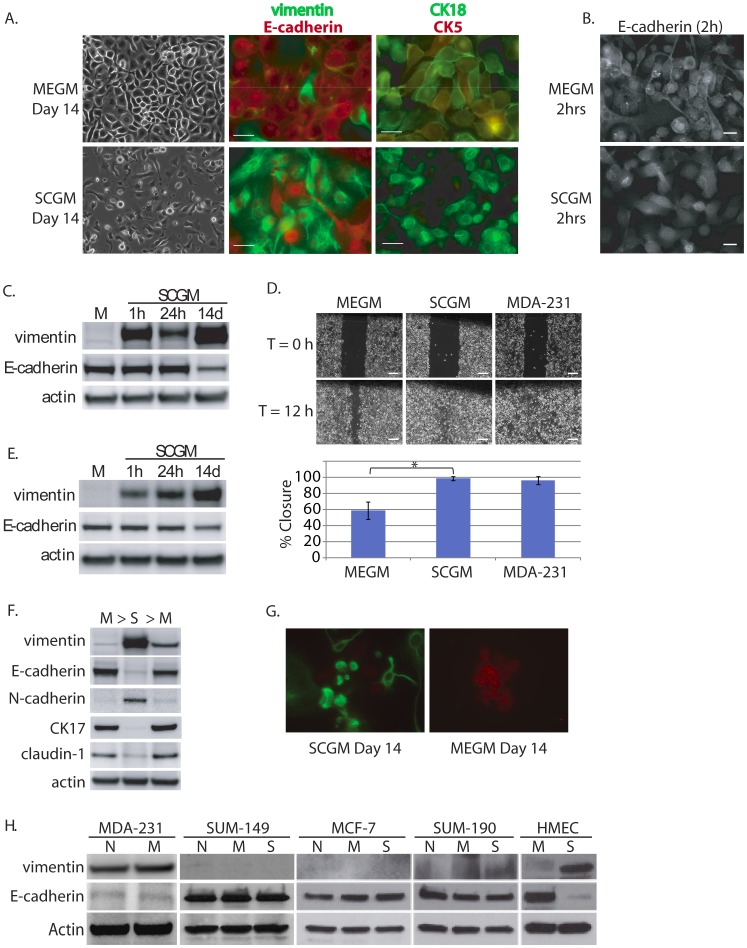
Evidence for morphologic and phenotypic changes consistent with *in vitro* EMT. **A.** Phase contrast or immunofluorescence images of DKAT cells cultured in either MEGM (upper) or SCGM for 14 days (lower). Phase contrast images were acquired with a 20 × objective, scale bar = 40 µm. IF images were acquired with a 40 × objective, scale bar = 20 µm. Cells were stained simultaneously for vimentin (green) and E-cadherin (red), or CK18 (green) and CK5 (red). **B.** E-cadherin immunofluorescence images of DKAT cells cultured in MEGM or SCGM for 2 hours. **C.** Total cell lysates from DKAT cells cultured in MEGM or SCGM for 1 h, 24 h, or 14 days were analyzed by western blot for vimentin, E-cadherin and actin. **D.** Scratch wound healing assay comparing DKAT cells grown in MEGM or SCGM (14 days) and MDA-MB-231 cells. Images (upper panel) were taken at the time of the scratch (T = 0) and 12 hours later with a 5 × objective. Scale bar = 200 µm. Results (lower panel) are expressed as the percentage of the scratch area closed at 12 hrs as measured by ImageJ software, and are an average of 12 scratches per sample. **E.** Total cell lysates from a clonal DKAT cell line cultured in MEGM or SCGM (14 days) were analyzed by western blot for vimentin, E-cadherin and actin. **F.** Total cell lysate from passage matched cultures of DKAT cells grown in MEGM, SCGM, or SCGM for 10 passages and then returned to MEGM for 5 passages were analyzed by western blot for epithelial and mesenchymal markers. **G.** Immunofluorescence (IF) images of subcloned DKAT-SCGM cells cultured in SCGM or MEGM for 14 days. Cells were stained simultaneously for vimentin (green) and E-cadherin (red), and images were acquired with a 40 × objective. **H.** Total cell lysates from the indicated cell lines cultured in their normal media or switched to MEGM or SCGM for 14 days were analyzed by western blot for vimentin, E-cadherin, and actin expression. N = Normal, M = MEGM, S = SCGM.

Western blot analysis demonstrated that at baseline, DKAT cells grown in Mammary Epithelial Growth Media (MEGM) expressed E-cadherin and a low level of vimentin (Figure 3B). Immunofluorescence studies showed that DKAT cells cultured in MEGM stained positive for the epithelial markers CK5, CK18, and E-cadherin, which was found both at the membrane and throughout the cytoplasm. Staining for the mesenchymal marker vimentin was weak and scattered (Figure 4A).

To induce *in vitro* EMT, DKAT cells were switched from serum-free MEGM to Stromal Cell Growth Media (SCGM) containing insulin, bFGF, and 5% FBS. In contrast to their epithelial phenotype when grown in MEGM, DKAT cells grown in SCGM for 14 days were predominately vimentin-positive and E-cadherin-negative, though a small percentage of cells retained an epithelial staining pattern (Figure 4A). Additionally, we observed a significant decrease in epithelial markers CK5 and CK18, consistent with a shift from expression of cytokeratin intermediate filaments to vimentin [29] (Figure 4A). While CK5 was uniformly downregulated, CK18 was still expressed at low levels in some of the cells. Strikingly, within 2 hours of being cultured in SCGM, DKAT cells underwent a morphological change to a more spindle-shaped and less cobblestone appearance (Figure 4B).

To assess expression of E-cadherin and vimentin at various time points after the switch to SCGM, DKAT cells were cultured in SCGM and protein lysate was collected 1 hour, 24 hours, or 14 days later. DKAT cells cultured in SCGM demonstrated a 100-fold increase in vimentin expression as early as 1 h, and expression of E-cadherin was strongly decreased by day 14 (Figure 4C). While we did not observe a rapid downregulation of E-cadherin protein by western blot following SCGM treatment, immunofluorescence demonstrated that within 2 hours of SCGM treatment, E-cadherin was predominately localized in the cytoplasm and away from the membrane (Figure 4B).

We next tested whether DKAT cells grown in SCGM demonstrated increased migration using a scratch/wound healing assay. DKAT cells grown in SCGM for 14 days migrated to fill the area of the scratch more quickly than DKAT cells in MEGM, with almost 100% closure by DKAT-SCGM cells at 12 hr versus 60% closure for DKAT-MEGM cells (Figure 4D). This increased migratory behavior further supports the mesenchymal phenotype of DKAT cells grown in SCGM.

It is possible that rather than inducing EMT, long-term culture in SCGM results in the selection of cells already in the mesenchymal state. To further investigate this possibility, we sub-cultured clonal populations derived from single DKAT cells in MEGM. We observed that one such clonal population with low vimentin and high E-cadherin levels in MEGM underwent the same vimentin upregulation and E-cadherin downregulation when cultured in SCGM, with similar kinetics as the bulk population of DKAT cells (Figure 4E).

To test for the ability of DKAT cells to undergo *in vitro* MET, we took DKAT cells that had been cultured in SCGM for 10 passages and exhibited a mesenchymal phenotype, and switched them back to growth in MEGM media. Five passages after being returned to MEGM, DKAT cells demonstrated a significant decrease in vimentin expression and increase in E-cadherin (Figure 4F), consistent with a reversion to the original epithelial phenotype. Western blotting also showed dramatically increased expression of the mesenchymal marker N-cadherin and decreased levels of epithelial markers CK17 and claudin-1 after 14 days in SCGM. Five passages after being returned to MEGM, N-cadherin expression was significantly decreased and CK17 and claudin-1 returned to their original expression levels (Figure 4F).

We also tested a clonal population derived from a single DKAT-SCGM cell with a mesenchymal phenotype for the ability to undergo *in vitro* MET. Following 14 days in MEGM, immunofluorescence for E-cadherin and vimentin showed that this clonal cell line contained cells with two predominantly two different phenotypes (Figure 4G). One population was morphologically cobblestone in appearance and stained positive for membranous E-cadherin and negative for vimentin. A second population displayed a more spindle-shaped morphology and was E-cadherin-negative and vimentin-positive. Taken together, these observations provide evidence that in response to altered culture conditions, DKAT cells undergo phenotypic and morphological changes consistent with EMT and MET.

In order to determine if the properties of the DKAT cell line are unique or whether growing any breast cancer line in MEGM or SCGM would result in similar phenotypic shifts, we cultured a variety of breast cell lines in MEGM or SCGM. MDA-MB-231 breast cancer cells are categorized as Basal B or mesenchymal according to gene expression profiling [28], [30], [31] and are frequently used to study breast cancer metastasis. These cells are typically cultured in medium containing 5–10% FBS, therefore we cultured this cell line in MEGM and looked for evidence of MET. After 14 days in MEGM, western blotting showed no change in the level of E-cadherin or vimentin protein expression, indicating MET had not occurred (Figure 4H).

Conversely, MCF-7, SUM-149, and SUM-190 breast cancer cells are normally grown in media containing 5–10% FBS and express epithelial markers. After culturing these three cell lines in SCGM for 14 days, we saw no evidence of EMT (Figure 4H). Furthermore, culturing these two cell lines in MEGM did not have an effect on the expression of E-cadherin or vimentin. Finally, we also tested the effect of growing hTERT-immortalized human mammary epithelial cells (HMECs) in SCGM for 14 days. While culturing these cells in SCGM did result in decreased E-cadherin and a small increase in vimentin (Figure 4H), the non-transformed cells did not proliferate in the presence of serum and arrested during the 14 day time course [32] (data not shown). These experiments demonstrate that the DKAT cell line is unique among the tested breast cancer models for its ability to reversibly transition between an epithelial or mesenchymal phenotype based on the culture media in which the cells are grown.

mRNA expression was compared in DKAT cells grown for 14 days in MEGM versus SCGM. As expected, DKAT cells grown in SCGM media demonstrated a significant decrease in epithelial-specific gene expression, including genes that regulate adhesion and cell-cell junctions. A significant increase in genes associated with a mesenchymal phenotype and cellular transformation was also observed. A partial list of differentially expressed genes is presented in Table 3. Of note, there was a decrease in expression of *KRT6C* (CK6; 131.8-fold), *KRT5* (CK5; 37.0-fold), *TP63* (p63; 26.2-fold), *KRT17* (CK17; 7.8-fold), *KRT14* (CK14; 3.0-fold), and *CLDN1* (claudin-1; 2.9-fold) and an increase in *TGFA* (TGF-alpha; 2.3-fold), *TGFBI* (TGF-beta-induced; 2.6-fold), *LOX* (lysyl oxidase; 2.9-fold), *ALDH1A3* (ALDH-1a3; 3.6-fold), *CDH2* (N-cadherin; 5.6-fold), and *FGF2* (FGF2; 6.0-fold). As shown in Figure 4F, western blotting for claudin-1, cytokeratin 17, and N-cadherin from protein lyastes of DKAT cells that had undergone EMT/MET as described above confirmed the results of the microarray data.

**Table 3:** Differential gene expression (MEGM/SCGM).

Fold Change(MEGM to SCGM)	Probe Set	Gene Symbol	Description
−131.8	213680_at	KRT6C	keratin 6C
−37.0	201820_at	KRT5	keratin 5
−26.2	209863_s_at	TP63	tumor protein p63
−8.8	214580_x_at	KRT6A	keratin 6A
−9.1	211194_s_at	TP63	tumor protein p63
−7.8	205157_s_at	KRT17	keratin 17
−6.7	204105_s_at	NRCAM	neuronal cell adhesion molecule
−4.4	208083_s_at	ITGB6	integrin, beta 6
−4.0	200953_s_at	CCND2	Cyclin D2
−3.0	209351_at	KRT14	keratin 14
−2.9	222549_at	CLDN1	claudin 1
−3.0	229041_s_at	ITGB2	integrin, beta 2
−3.0	204990_s_at	ITGB4	integrin, beta 4
−3.0	217312_s_at	COL7A1	collagen, type VII, alpha 1
−2.5	209270_at	LAMB3	laminin, beta 3
−3.0	211473_s_at	COL4A6	collagen, type IV, alpha 6
−2.4	215177_s_at	ITGA6	integrin, alpha 6
			
2.3	205016_at	TGFA	transforming growth factor, alpha
2.6	201506_at	TGFBI	transforming growth factor, beta-induced
2.7	205422_s_at	ITGBL1	integrin, beta-like 1
2.8	212489_at	COL5A1	collagen, type V, alpha 1
2.9	215446_s_at	LOX	lysyl oxidase
3.4	204726_at	CDH13	cadherin 13, H-cadherin
3.5	205885_s_at	ITGA4	integrin, alpha 4
3.6	203180_at	ALDH1A3	aldehyde dehydrogenase 1 family, member A3
3.9	226622_at	MUC20	mucin 20, cell surface associated
4.2	202016_at	MEST	mesoderm specific transcript homolog
5.6	203440_at	CDH2	cadherin 2, type 1, N-cadherin
5.7	224480_s_at	MAG1	lung cancer metastasis-associated protein
6.0	204422_s_at	FGF2	fibroblast growth factor 2 (basic)

List of genes related to the epithelial phenotype, cell-cell junctions, and motility with altered expression following 14 days of culture in SCGM to induce EMT. Microarray experiments were performed as described in Materials and Methods.

### Zeb1 Regulates DKAT Cell Plasticity

**Figure 5:**
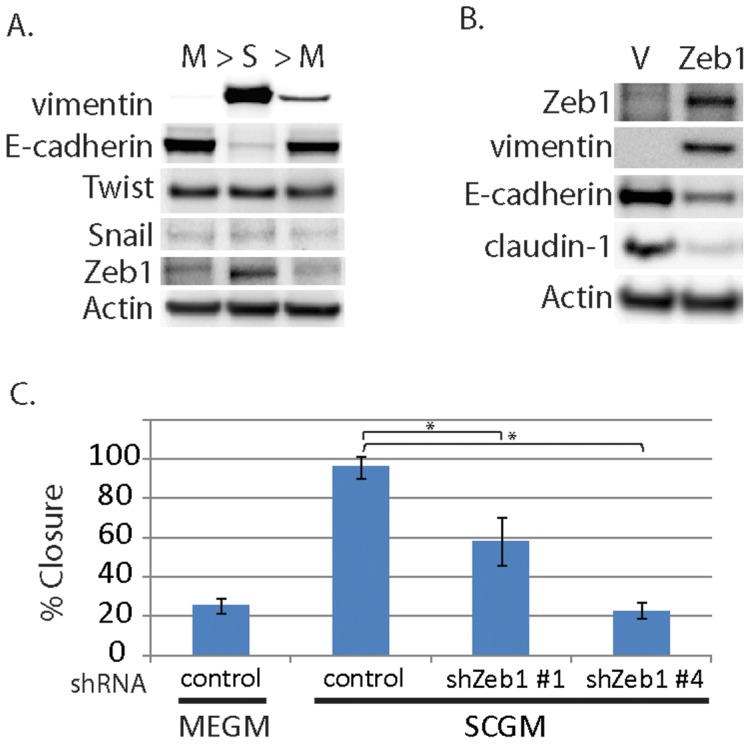
Zeb1 regulates DKAT EMT/MET. A. Total cell lysate was prepared from DKAT-MEGM or DKAT-SCGM cells and subjected to western blot analysis for markers of EMT. **B.** Total cell lysate was prepared from DKAT-MEGM cells stably transfected with a control vector (V) or vector containing Zeb1 transcript (Zeb1) and subjected to western blot analysis for markers of EMT. **C.** Scratch wound healing assay comparing DKAT cells grown in MEGM or SCGM as indicated, stably expressing either a non-targeting shRNA sequence or one of two Zeb1-targeting sequences. Results are expressed as the percentage of the scratch area closed at 16 hrs as measured by ImageJ software, and are an average of 12 scratches per sample.

A large number of transcription factors are implicated in the process of EMT, including members of the Snail, bHLH, and Zeb families, all of which have been reported to be capable of repressing E-cadherin and inducing EMT in various cell types [33]. In order to further investigate the mechanisms underlying epithelial plasticity in the DKAT cell line, we looked at the expression of three of these transcription factors in the DKAT model. Western blotting showed no noticeable difference in expression of Snail1 and Twist protein between DKAT cells grown in MEBM versus SCBM. However, Zeb1 was noticeably increased in DKAT-SCGM cells compared to DKAT-MEGM cells (Figure 5A). Zeb1 is a zinc-finger transcription factor that binds directly to the E-cadherin promoter and represses its transcription [34]. To determine if over-expression of Zeb1 is sufficient to induce phenotypic changes consistent with EMT, we constitutively expressed Zeb1 in epithelial DKAT-MEGM cells. Constitutive over-expression of Zeb1 resulted in a shift to a mesenchymal phenotype, evidenced by decreased expression of E-Cadherin and Claudin-1 and increased vimentin (Figure 5B). Notably, stable knockdown of Zeb1 using two different lentiviral shRNA sequences prevented the increase in migration when DKAT cells were switched from MEGM to SCGM (Figure 5C). Together, these data indicate that Zeb1 is important in both the induction of EMT in the DKAT model and the increased migratory ability of the mesenchymal DKAT cells.

### DKAT Cells are Highly Tumorigenic and Exhibit *in vivo* Plasticity

To test the tumorigenic potential of the DKAT cell line, 10^4^, 10^3^, 10^2^, or 10 cells grown in MEGM were injected into the mammary fat pad of immunocompromised mice. Palpable tumors were present within 2–4 weeks and most animals were sacrificed by 13 weeks post-injection with tumors of 15 mm diameter. We observed that 8/8 mice developed tumors at doses of 10^4^, 10^3^ and 10^2^ cells/injection. Importantly, 2/8 mice formed tumors when only 10 cells were injected, indicating the significant tumorigenicity of the DKAT cell line.

**Figure 6:**
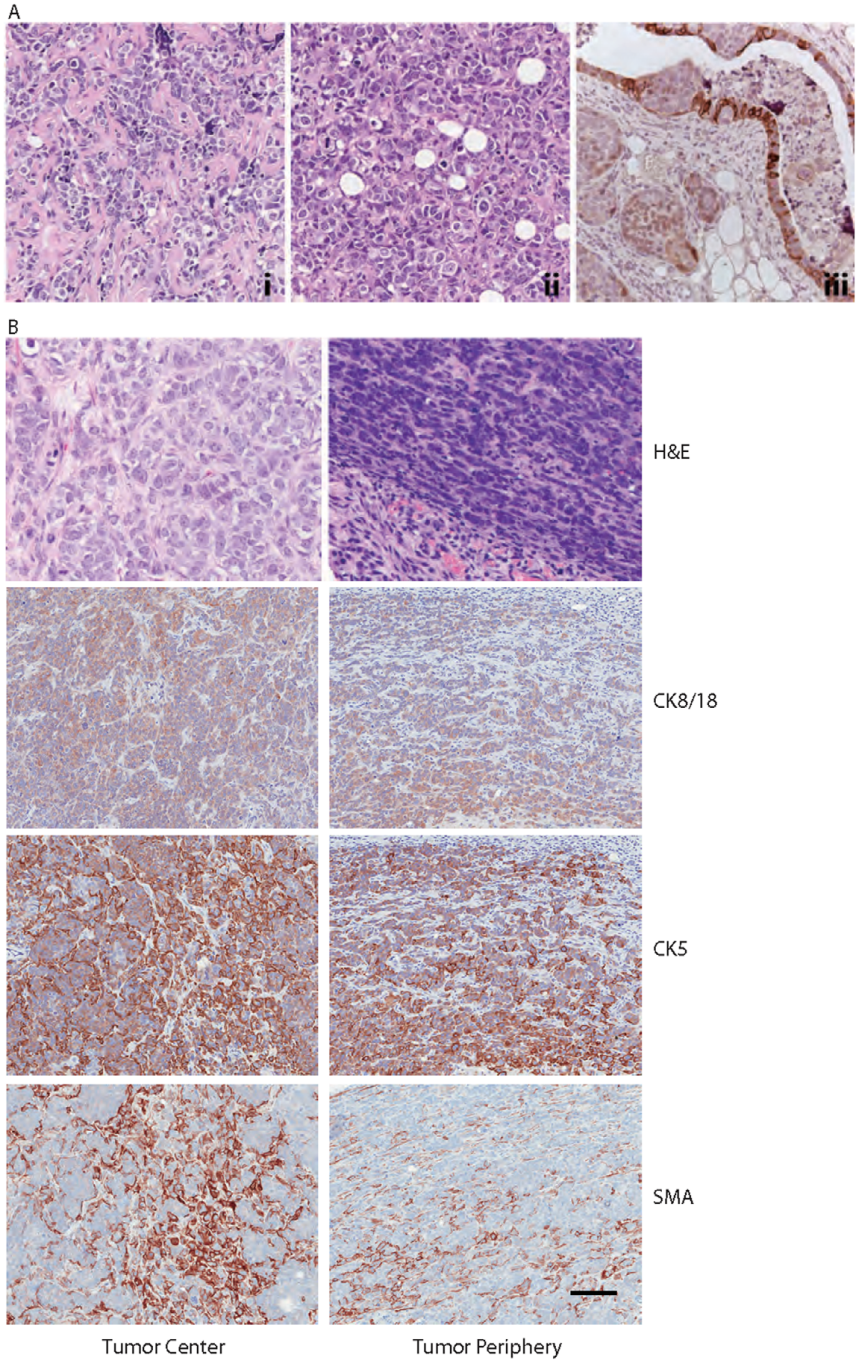
Comparison of H&E and IHC patterns from DKAT xenografts. **A.** IHC showing H&E (i and ii) or CK5 staining (iii) illustrating the three recurring morphological patterns observed in DKAT xenografts transplanted without Matrigel. **B.** IHC showing H&E, CK8/18, CK5, and SMA staining of DKAT xenografts transplanted with Matrigel. The left column shows the center of a transplant and the right column shows the leading edge of the tumor.

Similar to the human cancer of origin, DKAT xenograft tumors exhibited a range of morphological patterns and immunohistochemical staining. Three recurring morphological patterns were observed in DKAT xenografts transplanted without Matrigel. *Pattern I:* Infiltrating growth without dense fibrous matrix (Figure 6Ai). DKAT cells formed cords and columns of neoplastic cells that infiltrated the surrounding fat and stained positive for CK5. *Pattern II:* Infiltrating growth with dense fibrous matrix (Figure 6Aii). DKAT cells formed nests and cords of cells surrounded by a dense connective tissue. Notably, DKAT cells were observed inside the ducts of the host mammary gland demonstrating the ability of DKAT cells to invade through the basement membrane. *Pattern III:* Intraductal and Pagetoid spread (Figure 6Aiii). One xenograft was not palpable but was visible when the host fat pad was exposed. This transplant lay flat in the fat pad spreading along the ductal tree, but not forming a three-dimensional mass. The whole mount revealed an abnormal irregular branching pattern that is frequently found in outgrowths of mouse mammary intraepithelial neoplasia. In some areas, the tumor cells infiltrated as small nests between the myoepithelium and the luminal cells of the mouse (Pagetoid spread). In other areas, the human cancer cells were clearly invading the stroma without any recognizable association with mouse mammary cells. A dense inflammatory infiltrate surrounded all areas of the outgrowth. DKAT cells stained for CK5, but did not stain for vimentin.

The addition of Matrigel significantly affected the morphological and staining patterns of DKAT xenograft tumors. DKAT xenografts transplanted with Matrigel formed nests and cords of cells that stained for both CK5 and SMA (Figure 6B), suggesting the formation of myoepithelial cells. The peripheral cells infiltrated surrounding tissue and enclosed nests of epithelial cells that stained positive for CK8/18 and negative for SMA. The DKAT cells in the center of the tumor (Figure 6B, left column) have larger nuclei with open chromatin and more abundant cytoplasm. IHC for SMA and CK5 shows clusters of unstained cells surrounded by dual staining cells. In contrast, the invasive cells at the edge of the tumor (Figure 6B, right column) from cords of cells that are intensely positive for CK5 and some SMA.

**Figure 7:**
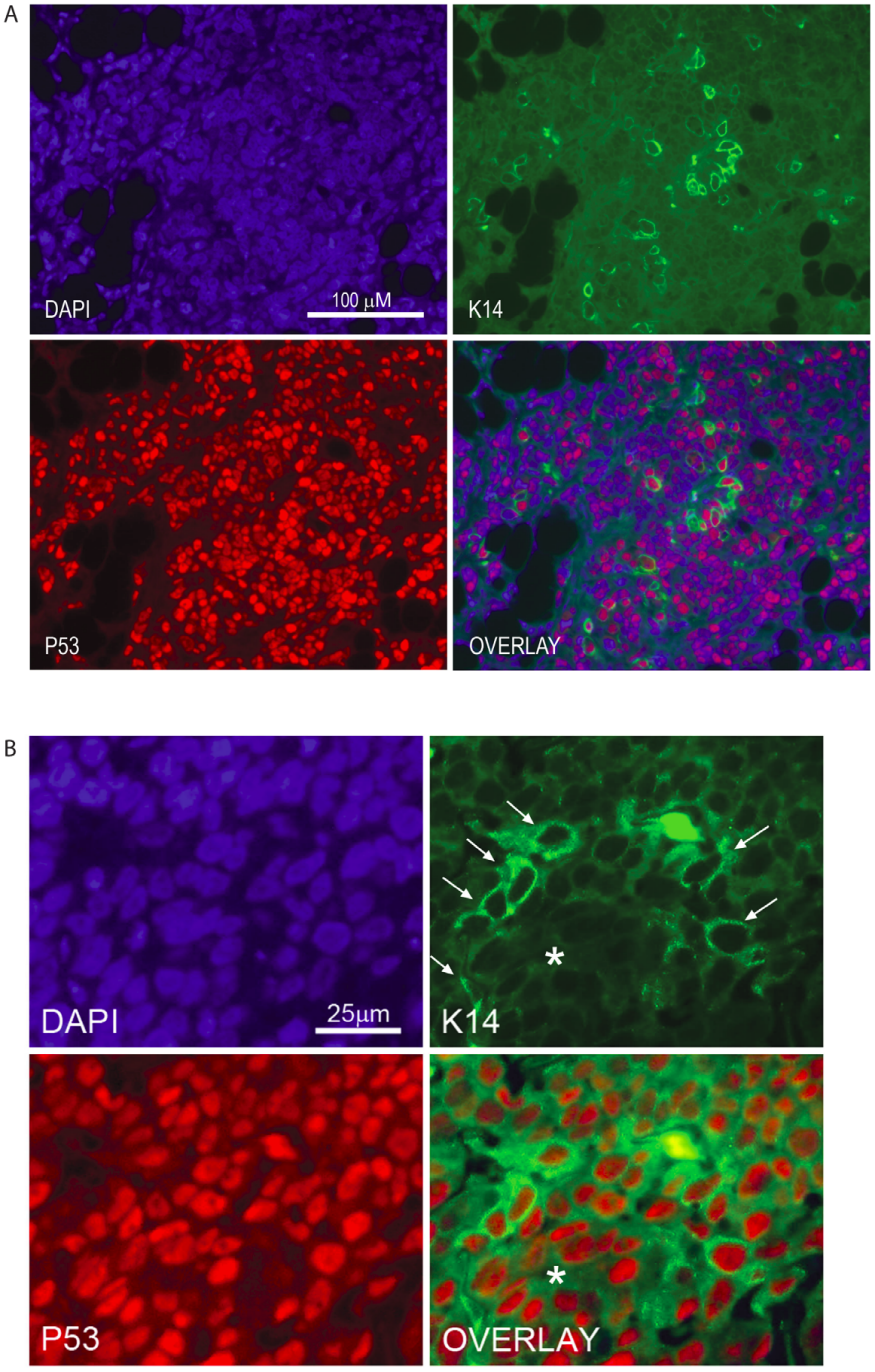
Basal/myoepithelial differentiation of DKAT xenografts. A. DKAT xenograft tumor stained with DAPI (blue) nuclear counter stain, CK14 (green) showing basal/myoepithelial differentiation of tumor cells, and human-specific p53 (red) confirming that both the CK14-positive cells and the more luminal, CK14 weak/negative cells are human DKAT tumor cells. 100 micron scale bar in the DAPI channel applies to all four images. **B.** DKAT tumor from a second mouse at higher magnification shows an area of vague glandular architecture with somewhat elongated CK14-positive basal/myoepithelial cells (arrows) surrounding CK14 weak/negative cells which surround a poorly formed glandular lumen (asterix). 25 micron scale bar show in in DAPI channel.

Three-color immunofluorescence staining (human-specific p53, CK14, and nuclear counter stain) was performed on the DKAT xenograft tumors to confirm that the observed expression of myoepithelial markers was due to heterogeneity of the DKAT tumor cells, rather than infiltration of host cells in the tumor microenvironment. The tumors contained human p53-positive cells with varying levels of CK14 expression, demonstrating that both the CK14-positive basal/myoepithelial cells and the more luminal CK14-weak/negative cells are human DKAT tumor cells (Figure 7A and 7B). There are also DAPI-positive cells that do not stain for human p53, likely representing admixed mouse cells. However, these cells are CK14 negative, confirming that the basal and myoepithelial differentiation in the tumor originates from the implanted DKAT tumor cells and not mouse fibroblasts or other host cells. Taken together, these results show that DKAT cells *in vivo* are invasive and exhibit phenotypic plasticity.

### Single DKAT Cells can Generate Tumorspheres Containing Both Epithelial and Mesenchymal Populations

**Figure 8:**
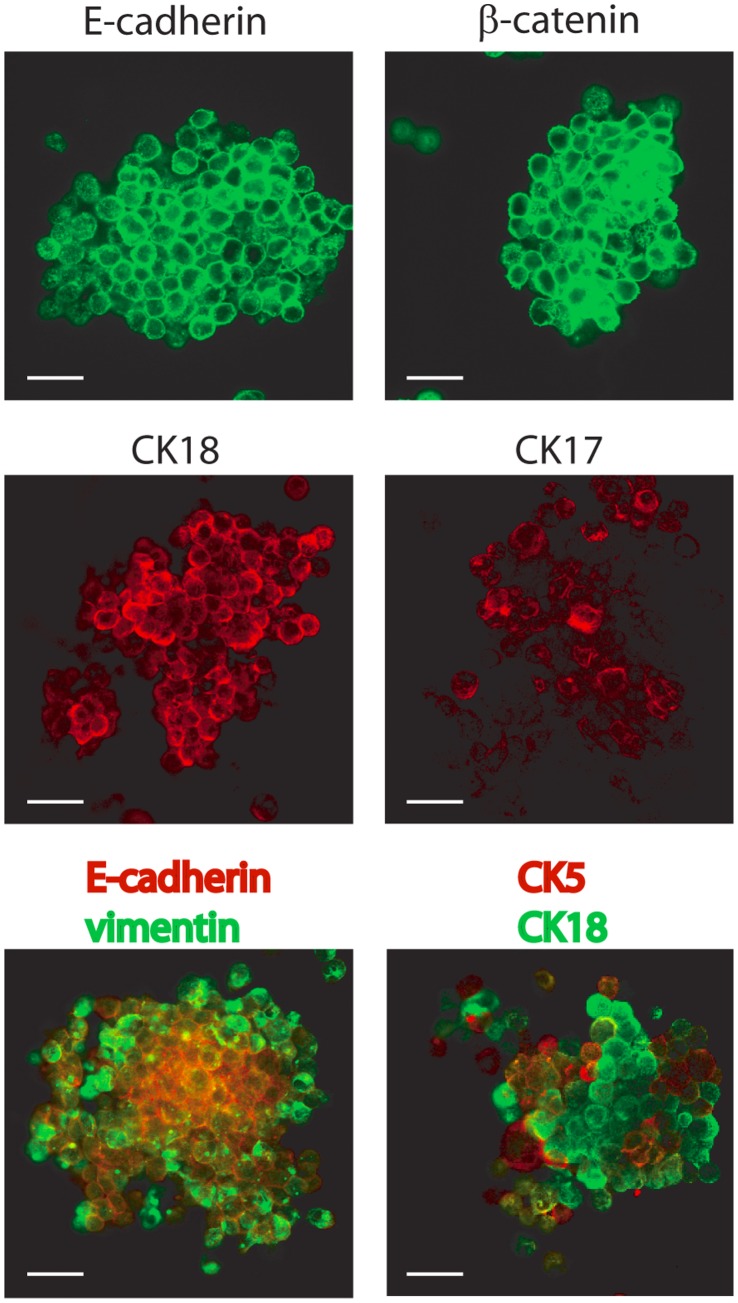
DKAT tumorsphere culture. Immunofluorescence of DKAT tumorspheres stained for E-cadherin, β-catenin, CK17 and CK18 (upper and middle panels). Tumorspheres were also dual stained for E-cadherin (red) and vimentin (green), or CK5 (red) and CK18 (green) (lower panel). Images were acquired with a 63 × objective. Scale bar = 40 µm.

We show that DKAT cells exhibit *in vitro* and *in vivo* plasticity. Because a number of recent studies have demonstrated a link between the EMT process and cancer stem cell properties, we tested whether a single DKAT cell was capable of forming multi-lineage cell clusters in non-adherent mammosphere culture conditions. DKAT cells readily formed solid mammospheres (tumorspheres) with a tumorsphere formation efficiency between 3 and 10%. Tumorspheres stained strongly for E-cadherin, β-catenin, CK5, CK17, and CK18 (Figure 8), stained weakly for SMA, and were negative for CK14 by immunofluorescence. Interestingly, the DKAT tumorspheres contained dual populations of cells that were either E-cadherin(+)/vimentin(−) or E-cadherin(−)/vimentin(+). Dual staining for CK5 and CK18 demonstrated that tumorspheres also contained populations of CK18(+)/CK5(low) and CK18(low)/CK5(+) cells (Figure 8). The presence of tumorspheres with dual epithelial and mesenchymal marker staining demonstrates that a single DKAT cell is able to generate both epithelial and mesenchymal populations in tumorsphere culture conditions.

## Discussion

Here we report the isolation of the DKAT cell line from a highly aggressive triple-negative human breast cancer that displayed morphological and biochemical characteristics suggestive of EMT and MET. The human breast cancer from which the DKAT cell line was isolated was comprised of multiple distinct foci, the majority of which displayed an epithelial staining pattern. However, one focus of the primary tumor, as well as the area of invasion into the chest wall, displayed a more mesenchymal phenotype, staining strongly for vimentin and diffuse, cytoplasmic E-cadherin (Figure 1). Interestingly, metastatic tumor cells in the bone marrow were found to have a staining pattern consistent with a reversion to an epithelial phenotype (Figure 1).

Based on morphology and immunostaining, it was not possible to determine whether this range of phenotypes was due to an outgrowth of cell populations with a fixed phenotype, or rather due to inherent phenotypic plasticity of the tumor cells. To explore these possible explanations, we tested DKAT cells for phenotypic plasticity *in vitro* using 2D and tumorsphere culture, and *in vivo* using mouse xenografts. DKAT cells in culture demonstrated evidence of phenotypic plasticity by reversible induction of vimentin and decreased E-cadherin in response to serum-containing media (Figure 4), and the formation of tumorspheres containing both epithelial and mesenchymal markers in 3D culture (Figure 8). Of the three most commonly-studied EMT-inducing transcription factors Snail1, Zeb1, and Twist, we found that only Zeb1 protein levels were increased during DKAT EMT, and this induction was necessary for the increased migratory ability of DKAT cells grown in SCGM (Figure 5).

An alternative explanation for the observed changes in DKAT cell morphology and EMT marker expression is that culture in SCGM selects for the outgrowth of a small pre-existing subpopulation of mesenchymal-like cells. In order to test for this possibility we generated clonal cell lines from single DKAT cells in MEGM. We found that these clonal lines generated from a single cell were able to undergo *in vitro* EMT with the same kinetics as the total DKAT population, providing evidence that this process is the result of plasticity inherent to the cells, rather than a long-term selection of a subpopulation of pre-existing mesenchymal cells (Figure 4D).

Furthermore, when a clonal SCGM-DKAT cell line derived from a single cell was cultured in MEGM, clusters of cells with membranous E-cadherin and low expression of vimentin were observed (Figure 4G). While not every cell underwent MET, these clonal cell line experiments demonstrate that a single DKAT cell is able to give rise to a population of cells capable of undergoing EMT and MET. Indeed it is likely that not all the cells in the DKAT population have the same potential for plasticity. The DKAT cell line is likely heterogeneous, just as primary tumor samples display heterogeneity among cells in their clonogenicity and stem cell characteristics.

A number of recent studies have demonstrated a link between EMT and the induction of stem cell-like characteristics in mammary epithelial cells [10], [27]. The DKAT cell line, which was isolated from a triple-negative tumor, contains a high percentage of CD44^hi^/CD24^−/(low)^ breast cancer stem-like cells (Figure 3C). Tumorsphere culture of single DKAT cells resulted in the growth of multi-lineage cell clusters with distinct populations of epithelial (both luminal and basal) and mesenchymal cells (Figure 8), further suggesting the inherent phenotypic plasticity of DKAT cells and supporting the idea that a stem-cell phenotype may be important in the epithelial plasticity of the cell line.

In our *in vivo* studies, similar to the multi-focal primary tumor from which the cell line was derived, we found that DKAT xenograft tumors exhibit a variety of phenotypes that appear to be dependent on the microenvironment and illustrate the plasticity of the DKAT cells (Figure 6). When placed into a standard subcutaneous environment, they invade into the surrounding tissue as cords of cells. When injected into an intact mammary fat pad, they are able to invade and colonize the host ducts. In Matrigel plugs, DKAT cells form nodules and nests with markers of both luminal and myoepithelial differentiation. Though traditional models of EMT involvement in tumor metastasis suggest that EMT would occur at the tumor periphery, we see SMA-positive DKAT cells in the center of the tumor. This is likely due to the presence of growth factors in the Matrigel used in the initial tumor cell injections. The *in vivo* plasticity of the DKAT cells suggests that the EMT/MET observed *in vitro* is not merely an artifact of the cell line adapting to culture conditions or selection of a subclone. Taken together, our data provide strong evidence that DKAT cells exhibit epithelial-mesenchymal plasticity, and that a single DKAT cell is capable of giving rise to cells with characteristics of multiple lineages.

Currently we are unable to reliably identify the subset of triple-negative breast cancers that are clinically aggressive and have the worst prognosis. Several new subtypes have been proposed within the triple-negative group based on gene expression patterns, including the Basal B, metaplastic, and claudin-low subtypes [18], [31], [35]. These marker classification systems are an important step forward in understanding the biology of aggressive breast tumors; however, the phenotypic plasticity of DKAT cells makes it difficult to precisely classify these cells by fixed marker expression studies and gene expression profiles. For example, DKAT cells induced to undergo *in vitro* EMT show reduced expression of tight junction proteins associated with the claudin-low phenotype; such classification may therefore be significantly influenced by culture conditions *in vitro* and the microenvironment *in vivo*.

While there has been intense interest in the study of EMT as it relates to breast cancer, there is still controversy over its contribution to cancer progression in patients. Without live image tracking or genetic tracing, the human tumor data presented here can only provide evidence of clonal heterogeneity. However, our *in vitro* experiments and *in vivo* xenograft studies demonstrate that a cell line derived from this cancer displays evidence suggestive of reversible EMT/MET and phenotypic plasticity. In both the total DKAT population and the clonal cell lines we observe that following the transition to SCGM, the acquisition of mesenchymal morphology and upregulation of vimentin are rapid events, evident within one hour. While E-cadherin is rapidly localized away from the membrane into the cytosol within 2 hours (Figure 4B) we do not observe decreased levels of total E-cadherin protein until after day 14. These observations are consistent with other reports that Zeb1 can inhibit expression of epithelial genes through recruitment of HDACs to target promoters, resulting in a repressive chromatin environment [36], [37]. This method of E-cadherin repression may partially explain the delayed reduction in E-cadherin protein levels, as well as the observed delay in re-expression of E-cadherin during the process of MET [36], [37].

Importantly, our results from the DKAT model of plasticity are consistent with clinical observations, namely that a full EMT is rarely seen in human cancer; rather, a partial EMT phenotype is most commonly observed in which cells exhibit both epithelial and mesenchymal traits simultaneously. The relevance of our studies to human cancer is underscored by recent data showing circulating tumor cells (CTCs) that stain positively for cytokeratin, vimentin, and N-cadherin were found in the majority of men with castration-resistant metastatic prostate cancer and women with metastatic breast cancer [38]. This intermediate epithelial-mesenchymal staining pattern of the CTCs suggests that epithelial plasticity, even within a small percentage of the tumor cell population, may be a driving factor in metastasis.

Traditional models of invasion and metastasis hypothesize that metastatic breast cancer results from the outgrowth of a subpopulation of cells with defined morphology and gene expression patterns, and that these cells are unique in their ability to migrate away from the primary tumor and establish a distant metastasis [39]. It has also been suggested that the aggressive metastatic behavior of certain subtypes of breast cancer (i.e. triple-negative) may reflect properties of the cell of origin from which the cancer arose [40], and more recently that the process of EMT itself may endow cancer cells with stem cell-like properties, enhancing their metastatic potential [10], [27], [41]. The ability of the DKAT cells to undergo reversible EMT in response to changes in culture conditions and their ability to form xenograft tumors with regions of both epithelial and mesenchymal characteristics provides evidence that the aggressive behavior of a subset of triple-negative breast cancers may be driven by the inherent phenotypic plasticity of the primary tumor. The DKAT model represents a unique triple-negative tumorigenic epithelial cell line that can undergo both EMT and MET in culture and is well suited for studies of the molecular mechanisms that regulate phenotypic plasticity in triple-negative breast cancer.

## Materials and Methods

### Establishment of the DKAT Cell Line

Pleural fluid from a patient with biopsy-confirmed primary breast cancer was obtained with the approval of The Ohio State University Institutional Review Board and with the written informed consent of the patient. Pleural fluid was centrifuged to pellet the cells. The pellet was then resuspended in Mammary Epithelial Cell Growth Medium (MEGM) supplemented with bovine pituitary extract, insulin, human recombinant epidermal growth factor, and hydrocortisone (Lonza, Basel, Switzerland). Mycoplasma testing was as previously reported [42].

### Other Cell Lines

Primary human mammary epithelial cells (HMECs) (Lonza) were immortalized with hTERT (HMEC-hTERT), and maintained in supplemented MEGM. MDA-MB-231 and MCF-7 cells (American Type Culture Collection [ATCC], Manassas, VA) were maintained in αMEM (Invitrogen, Carlsbad, CA) supplemented as previously described [43]. SUM-190 cells (ATCC) were maintained in RPMI supplemented with 10% FBS.

### Cytogenetic Analysis

Spectral karyotyping analysis (SKY) was performed as previously described [44]. Karyotypic abnormalities were classified according to the International System for Human Cytogenetic Nomenclature.

### Immunohistochemisty

The human formalin-fixed, paraffin-embedded primary breast biopsy, chest wall recurrence, and bone metastasis samples were sectioned at 4 µm thickness and stained for alpha-smooth muscle actin (1A4, Sigma), vimentin (3B4 Boehringer Mannheim Roche, Hvidovre, Denmark), CK5 (OVTM, DAKO, Glostrup, Denmark) and wide range keratins (MNF116, DAKO). The antibodies were visualized by streptavidin-biotin (DAKO 5004).

### *TP53* and *PIK3CA* Sequencing

Sequencing of exons 5–9 of the *TP53* gene and exons 9 and 20 of *PIK3CA* using previously published primers and programs [IARC TP53 Mutation Database, http://www-p53.iarc.fr/p53sequencing.html and [45]] was performed on genomic DNA isolated from passage 20 DKAT cells. Mutations were tested in both the sense and antisense sequences and were confirmed by repeat sequencing of a second, independent genomic DNA preparation.

### Immunofluorescence

Cells were fixed in either methanol for 20 minutes or 4% paraformaldehyde for 30 minutes on ice. Cells were blocked for 30 minutes, incubated with primary antibody overnight at 4°C and secondary antibody for 1 hour at RT, and counter stained with DAPI. Images were acquired on a Zeiss Axio Observer A1 fluorescence microscope (Carl Zeiss, Göttingen, Germany) with a 40 × or 63 × objective. Primary antibodies were from 1) Santa Cruz Biotechnology Inc. (Santa Cruz, CA): CK5 (sc-66856), CK14 (sc-53253), CK18 (sc-28264), CK17 (sc-101931), E-cadherin (sc-7870), EGFR (sc-120), ERalpha (sc-8005), p63 (sc-56188), and vimentin (sc-5565) 2) BD biosciences (San Jose, CA): vimentin (550513), E-cadherin (610181) 3) Thermo Fisher Scientific Inc. (Fremont, CA): PR (MS-298) 4) Abcam (Cambridge, MA): smooth muscle actin (ab15734), and 5) Invitrogen (Carlsbad, CA): claudin-1 (37–4900), claudin-4 (32–9400), ZO-1 (40–2300), occludin (40–4700), goat anti-mouse Alexa-fluor 488 (A21222) and goat anti-rabbit Alexa-fluor 597 (A11070) secondary antibodies.

### Western Blotting

Antibodies were p53 (DO-1, Santa Cruz Biotechnology Inc., Santa Cruz, CA); PTEN, Akt-pSer473, Akt-1, Akt-2, Akt-3 (9552, 4051 and 4058, 2967, 2964, 3788, respectively, Cell Signaling, Danvers, MA); claudin-1, claudin-4, occludin, ZO-1 (37–4900, 32–9400, 40–4700, and 40–2300, respectively; Invitrogen, Carlsbad, CA), snail-1 (gift from Antonio Garcia-deHerreros). Loading control was provided by reprobing the membrane with an antibody to beta-actin (I19, Santa Cruz Biotechnology, Inc.). Images were captured using the Kodak Image Station 2000 MM and quantified using Kodak 1D Image Analysis Software (Eastman Kodak, Rochester, MN).

### FACS Analysis

Cells were harvested with cell dissociation buffer (Invitrogen) and incubated with APC-conjugated CD44 and PE-conjugated CD24 antibodies (BD Biosciences, San Jose, CA). Fifty thousand events were collected using a FACSCaliber flow cytometer and analyzed for CD44/CD24 expression with CellQuest software (BD Immunocytometry Systems).

### Differential Gene Expression Studies

mRNA was collected using the Qiagen RNeasy mini kit with the optional DNAse step (Quiagen, Valencia, CA) and RNA integrity was confirmed by electrophoresis. cRNA synthesis and probe generation for cRNA array hybridization were performed according to the standardized protocols provided by Affymetrix™ (Affymetrix, Santa Clara, CA). Transcript analysis was performed using Affymetrix HG-U133 Plus2.0 GeneChips. Samples were labeled and hybridized following strict adherence to Affymetrix’s standardized protocols. GeneChip results were assessed for quality prior to further analysis. Median expression values of technical replicates (n = 3) for each of three cell lines, HMECs, DKAT, and MDA-MB-231, were compared with published breast cancer cell line expression data downloaded from the publisher’s web site [28]. Probe Set IDs were used to merge the two data sets (16,383 features). Duplicate Gene Symbols were summarized by mean Probe Set ID values. The data were array- and gene-wise median centered and filtered to exclude genes with standard deviations of observed values less than 1.5. The remaining 473 features were clustered using centroid linkage and Pearson correlation as the similarity metric (Cluster ver. 3.0). Data has been submitted to the NCBI GEO in accordance with MIAME guidelines.

### Migration Assays

5×10^5^ DKAT or MDA-MB-231 cells were plated in 6-well plates and cultured until confluent. The cell monolayer was then scratched with a pipette tip, rinsed, and photographed at regular intervals until the scratch was completely closed. The area of the scratch was measured using ImageJ software [46], and results are expressed as a percentage of the original scratch area filled by the cells at the indicated timepoint.

### *In vitro* Plasticity

EMT: DKAT cells were switched from MEGM to SCGM (Lonza); DKAT control cells remained in MEGM. Time in SCGM ranged up to 10 passages, and control DKAT cells were maintained in MEGM and passaged in parallel. MET: After 10 passages in SCGM, a portion of DKAT cells were switched back to MEGM. Control cells included 1) the original DKAT cells grown continuously in MEGM and 2) DKAT cells maintained in SCGM. All cells and controls were passaged in parallel and imaged and harvested at similar confluency.

### Clonal Cell Line Generation

DKAT cells in MEGM were digested into a single-cell suspension with trypsin and plated at one cell per well in a 96 well plate, and wells were visually checked for the formation of colonies. After 22 days, cells were removed from wells containing colonies by cell dissociation buffer (Invitrogen) and sequentially grown out in a 24-well plate, 6-well plate, T25 flask, and T75 flask. *In vitro* EMT was induced as previously described.

### Zeb1 Expression

The full-length Zeb1 transcript was amplified from MDA-MB-231 cells by PCR using the following primers: Forward 5′-CAA GCG AGA GGA TCA TGG CG-3′ and Reverse 5′-TTC CTT CTA GAA AAA CGA TTA GGC-3′. The resulting product was cloned into the XhoI and BamHI sites of pLXSN. Retroviral particles were generated by transfecting the resulting construct or the pLXSN empty vector control into GP293 cells. DKAT-MEGM cells were then transduced and expressing cells were selected with 0.5 mg/ml G418 (Invitrogen).

### Zeb1 shRNA

Mission shRNA constructs targeting Zeb1 and a non-targeting control construct were purchased from Sigma, and lentivirus was produced according to manufacturer’s instructions. DKAT cells in MEGM were transduced and selected with 0.5µg/ml Puromycin, and the resulting cells were grown in either MEGM (control) or SCGM to induce EMT. Scratch assays were performed at least 14 days after the transition to SCGM.

### Xenograft Experiments

DKAT cells grown in MEGM were tested for xenograft growth by limiting dilution; 10^4^, 10^3^, 10^2^, or 10 cells were resuspended in 50 µl of either 1) 1∶1/v:v PBS:Matrigel™ or 2) PBS alone, and injected into the right and left uncleared #4 mammary fat pad of NOD.CB17-PrkdcSCID/J mice. Four independent experiments (eight replicates per experiment) were performed at Duke University and University of California at Davis (UCD). DKAT xenografts using matrigel were performed with NOD CB17-Prkdc SCID/J mice (Jackson Labs) and were performed in accordance with a protocol approved by the Duke University Institutional Animal Care and Use Committee (protocol number A328-08-12). Tumor growth was assessed weekly by measuring tumor volume by caliper. Mice were sacrificed when the tumor reached 1.5 cm in diameter or after 150 days. Tumors were excised, fixed in 4% paraformaldehyde at 4°C overnight, sectioned and stained, and underwent central pathology review at UCD. DKAT xenografts without matrigel were performed in nude and NOD CB17-Prkdc SCID/J mice (Jackson Labs) in accordance with a protocol approved by the UCD Institutional Animal Care and Use Committee (protocol number 13217). All efforts were made to minimize suffering.

### Xenograft Immunofluorescence Staining

Three color (p53, keratin 14, nuclear counterstain) immunofluorescence staining was performed using standard protocols. Briefly, fresh frozen paraffin embedded 4 micron sections were mounted on positively charged slides, deparaffinized with xylene and washed in absolute ethanol. Endogenous peroxidase was quenched, and tissue was rehydrated prior to antigen retrieval (citrate, pH = 6.0, 2 minutes at 95°C). Tissue was blocked with Serum-Free Block (DAKO X090) for 15 minutes, then incubated at 4°C overnight with primary antibodies, 1∶500 anti-p53 goat polyclonal (Santa Cruz FL-393-G), 1∶200 anti-keratin 14 rabbit polyclonal (Thermo Scientific RB-9020-P1) in PBS with ovalbumin. After washing 5 minutes twice in PBS at room temperature, the tissue was first incubated in 1∶500 donkey anti-rabbit conjugated with Alexa Flour 488 (Invitrogen A21206) in PBS at room temperature for 30 minutes. After washing again 5 minutes twice in PBS, the tissue was incubated in 1∶500 rabbit anti-goat conjugated to Rhodamine (Millipore AP106R) in PBS at room temperature for 30 minutes, then washed twice in PBS for 5 minutes and coverslipped using DAPI Vectashield (Vector Labs), sealed with clear nail-polish and imaged. Images were acquired on a Zeiss Axiophot fluorescent microscope equipped with band-pass filters for FITC, DAPI, and Rhodamine. Images were obtained using a cooled CCD Zeiss color AxioCam and software. Image overlays were produced in Photoshop (Adobe).

### Tumorsphere Assays

Tumorsphere culture was performed as previously described [47] with minor modifications. Briefly, cells were plated at 1 cell/well in 96-well ultra-low attachment plates in MEGM containing B27, Heparin, EGF, and bFGF. Cells were cultured for 10–14 days and the tumorsphere formation efficiency was determined by counting the number of tumorspheres and dividing by the number of wells seeded. For immunofluorescence studies, DKAT cells were plated at 10^3^ cells/100 mm dish. Ten-14 days later, tumorspheres were pooled and fixed in 100% ice cold methanol, and staining was performed as described above. Tumorsphere images were acquired with an SP5 Leica laser scanning confocal microscope.

## Supporting Information

Figure S1 **Differential gene expression studies.** Median expression values of technical replicates (n = 3) for each of three cell lines, HMECs, DKAT, and MDA-MB-231 (orange) were analyzed by unsupervised hierarchical clustering with a published data set [28]. The results were displayed using Java TreeView (ver. 1.1.0). Cell lines are labeled by breast cancer subtype: luminal-like (blue), basal-like (red), mesenchymal-like (green), or unknown subtype (black).
(PDF)
