# Supplementary figures and images for: Evidence for Phenotypic Plasticity in Aggressive Triple-Negative Breast Cancer: Human Biology Is Recapitulated by a Novel Model System
Source: PLoS One. 2012 Sep 25;7(9):e45684. doi: 10.1371/journal.pone.0045684 (PMC3458110; doi:10.1371/journal.pone.0045684)

Figure S1

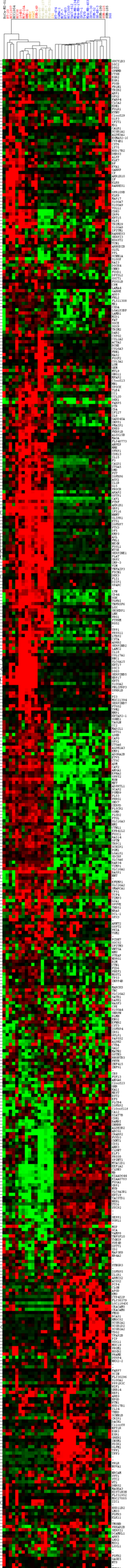

Supplement: Figure S1 — Differential gene expression studies. Median expression values of technical replicates (n = 3) for each of three cell lines, HMECs, DKAT, and MDA-MB-231 (orange) were analyzed by unsupervised hierarchical clustering with a published data set [28]. The results were displayed using Java TreeView (ver. 1.1.0). Cell lines are labeled by breast cancer subtype: luminal-like (blue), basal-like (red), mesenchymal-like (green), or unknown subtype (black). (PDF) [file pone.0045684.s001.pdf]
